# Prevalence and Outcomes of Sepsis in Patients With Colon Carcinoma: Organism-Specific Analysis

**DOI:** 10.7759/cureus.74175

**Published:** 2024-11-21

**Authors:** Rabia Iqbal, Zaigham ul Islam, Ahmad Taimoor Bajwa, Yaqub Nadeem Mohammed, Henry Kimball, Syeda Daniya Samreen, Qamar Iqbal, Sripada Preetham Kasire, Asmat Ullah

**Affiliations:** 1 Medicine, The Brooklyn Hospital Center, New York, USA; 2 Internal Medicine, Nishtar Medical University, Multan, PAK; 3 Internal Medicine, The Brooklyn Hospital Center, New York, USA; 4 Internal Medicine, Ascension Borgess Hospital, Kalamazoo, USA; 5 Internal Medicine, Trinity Health Oakland Hospital, Pontiac, USA; 6 Medicine, TidalHealth, Salisbury, USA; 7 Internal Medicine, New York City Health and Hospitals, New York, USA

**Keywords:** cancer-specific outcome, outcomes of colon cancer, sepsis and shock physiology, sepsis-related organ failure assessment, staphylococcus aureus bacteremia

## Abstract

Background

Sepsis is a challenging condition, especially in patients with malignancy, that is associated with worse mortality and increased complications. This study aimed to analyze the prevalence of sepsis, its complications, healthcare outcomes, and associated organism-specific mortality in patients with colorectal carcinoma using the National Inpatient Sample database.

Methodology

We included patients aged >18 years with a primary diagnosis of colon cancer. The patients were divided into two groups, those with sepsis and those without sepsis. Confounders were adjusted using multivariate regression analysis. We examined outcomes including mortality, hospital charges, length of stay, and other associated complications.

Results

Out of 876,769 patients diagnosed with colon cancer, 2,579 (0.2%) had methicillin-resistant *Staphylococcus aureus* (MRSA) sepsis, 1,004 (0.1%) had *Staphylococcus aureus* sepsis, and 6,439 (0.7%) had *Escherichia coli* sepsis. Patients with sepsis exhibited significantly longer hospital stays, the highest with *Staphylococcus aureus* and *Enterococcus*, as well as increased healthcare costs compared to those without sepsis. The highest mortality rates were associated with *Staphylococcus aureus* (n = 169/1,004, 17%), followed by *Pseudomonas* (n = 150/940, 16%) and MRSA (n = 374/2,579, 14%). Sepsis also led to higher rates of complications, including acute kidney injury and septic shock.

Conclusions

Through this study, we aim to highlight the need for early diagnosis and targeted management of colon cancer patients who develop sepsis during their hospital course. Future research should focus on the underlying pathophysiology and effective interventions to improve outcomes for patients with colorectal carcinoma and sepsis.

## Introduction

Sepsis is defined as “an uncontrolled host response to infection causing life-threatening organ damage” [1]. It is a major clinical challenge and is associated with increased mortality. The mortality rate ranges from 10% to 52% depending on different populations and data collection methods [2-7].

Malignancy is a significant risk factor for the development of sepsis. Patients with cancer are at 10 times higher risk of sepsis than the general population [8]. It can be attributed to several factors, such as immunosuppression due to underlying disease, chemotherapy, radiotherapy, and invasive procedures such as urinary and central venous catheters [9].

Colorectal carcinoma is the third most common type of cancer diagnosis and the second most common cause of cancer-related deaths in the United States [10]. Severe abdominal infection and sepsis are serious complications that can occur in patients with colorectal carcinoma and negatively impact the clinical outcomes. Interestingly, the disturbance is in the gut microflora, and infections are associated with the pathogenesis of colorectal carcinoma [10,11].

Despite the known risk, the prevalence of sepsis and its effect on prognosis and healthcare outcomes in patients with colon cancer has not been studied extensively. Minimal data are available regarding the prevalence and consequences of sepsis in colon cancer patients. To fill this gap, we conducted a retrospective analysis using the National Inpatient Sample (NIS) database to study the prevalence of sepsis, its complications, and its effect on hospital utilization cost, healthcare outcomes, and mortality in patients with colorectal carcinoma. A better knowledge of the relationship between sepsis and colorectal carcinoma can drive better healthcare decisions and overall outcomes in this patient population.

## Materials and methods

Data source and study population

The study used the NIS database from 2017 to 2020. NIS is the most extensive database maintained by Healthcare Cost and Project (HCUP), containing information about patient inpatient hospital stays. It provides information about patients’ demographics, diagnoses, complications, and outcomes. This study did not require ethical approval as the information was de-identified and publicly available.

The study included patients with a primary diagnosis of colon cancer. We used the International Classification of Diseases 10th Revision, Clinical Modification (ICD-10-CM) to detect patients with the diagnosis. All patients aged >18 were included.

The NIS database is a large, nationally representative dataset collected from various hospitals across the United States. It includes information about discharge from hospitals across the United States and allows us to capture data from multiple institutions. Although the authors from across these institutions do not interact directly, NIS provides comprehensive data representing diverse healthcare settings, which helps authors perform multi-institutional studies.

Study variables and outcomes

We collected data regarding patients’ demographics, including age, gender, and race. We also collected data regarding common comorbidities of colon cancer patients. We then examined the prevalence of sepsis and outcomes among colon cancer patients. The organisms studied causing sepsis included methicillin-resistant *Staphylococcus aureus* (MRSA), *Staphylococcus aureus*, *Escherichia coli*, *Enterococcus*, *Pseudomonas*, and other gram-negative organisms. The primary outcomes studied included mortality and hospital resource utilization. The secondary outcomes included acute kidney injury, peritonitis, gastrointestinal bleeding, septic shock, and altered mental status.

Statistical analysis

Stata version 17 (StataCorp., College Station, TX, USA) was used for data analysis. The categorical variables (complications) were analyzed using the chi-square test, and continuous variables (length of stay and total charges) were analyzed using the t-test. We also adjusted for the confounders, including smoking, alcohol intake, hypertension, dyslipidemia, chronic liver disease, chronic kidney disease, and chronic obstructive pulmonary disease. Univariate logistic regression was used to calculate the unadjusted odds ratio. Multivariate logistic regression was used to analyze the adjusted odds ratio (aOR). P-values <0.05 were considered significant.

## Results

Baseline demographics

A total of 876,769 patients were detected with the diagnosis of colon cancer in the years 2017 to 2020. The mean age of the patients was 67 years. Table 1 presents the demographic characteristics of the study population.

**Table 1:** Baseline demographics of patients with and without sepsis.

Demographics	With sepsis	Without sepsis	P-value
Methicillin-resistant *Staphylococcus aureus* infection			
Age (in years)	65	67	0.01
Gender			
Male	63%	52%	0.03
Female	37%	48%	0.03
Race			
White	72%	70%	0.3
Black	12%	14%	0.3
Hispanic	8%	9%	0.3
Staphylococcus aureus			
Age (in years)	66	67	0.01
Gender			
Male	53%	52%	0.8
Female	47%	48%	0.8
Race			
White	65%	70%	0.6
Black	17%	14%	0.6
Hispanic	12%	9%	0.6
Escherichia coli			
Age (in years)	67	67	0.01
Gender			
Male	55%	51%	<0.01
Female	45%	49%	<0.01
Race			
White	60%	70%	<0.01
Black	16%	14%	<0.01
Hispanic	15%	10%	<0.01
Pseudomonas			
Age (in years)	66	67	0.01
Gender			
Male	62	52	0.01
Female	38	48	0.01
Race			
White	71%	69%	0.8
Black	14%	14%	0.8
Hispanic	1%	10%	0.8
Enterococcus			
Age (in years)	67	67	0.01
Gender			
Male	57%	51%	0.05
Female	43%	49%	0.05
Race			
White	68%	70%	0.6
Black	12%	14%	0.6
Hispanic	10%	10%	0.6
Gram-negative organisms			
Age (in years)	66	67	0.03
Gender			
Male	55%	52%	0.1
Female	45%	48%	0.1
Race			
White	65%	70%	0.06
Black	15%	14%	0.06
Hispanic	11%	11%	0.06

Prevalence of sepsis

A total of 876,769 patients with colon cancer were detected. Of them, 2,579 (0.2%) patients had MRSA sepsis, 1,004 (0.1%) patients had *S. aureus* sepsis, 6,439 (0.7%) patients had *E. coli* sepsis, 940 (0.1%) patients had *Pseudomonas* sepsis, 1,935 (0.1%) had *Enterococcus* sepsis, and 3,090 (0.3%) patients had gram-negative sepsis.

Hospital resource utilization

Length of stay was increased in patients whose hospital stay was complicated by sepsis. The mean length of stay was increased in sepsis caused by MRSA (12 vs. 6.5 days, p < 0.05) and *E. coli* (11 vs. 6.5 days, p < 0.05) (Table 2).

**Table 2:** Total length of stay for patients with and without sepsis categorized by organisms.

Organism	With sepsis	Without sepsis	Adjusted odds ratio	P-value
Methicillin-resistant *Staphylococcus aureus*	12	6.5	2.9	0.01
Staphylococcus aureus	14	6.5	1.2	0.7
Escherichia coli	11	6.5	3.1	0.01
Pseudomonas aeruginosa	13	6.5	3.2	0.1
Enterococcus	14	6.5	1.5	0.4
Other gram-negative organisms	11	6.5	3.6	0.06

As shown in Table 3, the total hospital charges were also increased when the hospital admission was complicated by sepsis.

**Table 3:** Total hospital charges (US dollars) for patients with and without sepsis categorized by organisms.

Organisms	Total hospital charges with sepsis (US dollars)	Total hospital charges without sepsis (US dollars)	P-value
Methicillin-resistant *Staphylococcus aureus*	147,789	78,761	0.00
Staphylococcus aureus	150,181	7,883	0.00
Escherichia coli	138,153	78,527	0.00
Pseudomonas aeruginosa	170,432	78,866	0.00
Enterococcus	193,551	78,712	0.00
Other gram-negative organisms	133,769	78,771	0.00

Mortality

The presence of sepsis increased mortality in colon cancer patients. The highest rate of mortality was observed in patients with *S. aureus* sepsis (17%), followed by *Pseudomonas* (16%) and MRSA (14%). The results were significant (p = 0.00). Univariate and multivariate logistic regressions for the mortality of all the organisms are shown in Table 4.

**Table 4:** Mortality of the patients with and without sepsis categorized by organisms.

Organism	With sepsis (N)	With sepsis (%)	Without sepsis (N)	Without sepsis (%)	Unadjusted odds ratio	P-value	Adjusted odds ratio	P-value
Methicillin-resistant *Staphylococcus aureus*	374	14%	41,654	4%	3.3	0.00	3.1	0.00
Staphylococcus aureus	169	17%	41,869	5%	4.0	0.00	3.8	0.00
Escherichia coli	700	10%	41,329	4.7%	2.4	0.00	2.1	0.00
Pseudomonas	150	16%	41,879	4.7%	3.7	0.00	3.2	0.00
Enterococcus	214	6.4%	42,814	4.7%	2.4	0.00	2.1	0.00
Other gram-negative organisms	349	11.2%	41,679	4.7%	2.5	0.00	2.4	0.00

Complications

The complications in colon cancer patients with and without sepsis were also evaluated. The presence of sepsis caused higher complications compared to patients without sepsis, as shown in Table 5. Acute kidney injury was the most common among all complications, the highest being among sepsis caused by *Enterococcus* (52% vs. 18% in patients without sepsis, p < 0.00). Peritonitis was increased in *E. coli* and *Enterococcus* groups (p < 0.05), while it was not significant in sepsis caused by other organisms. Sepsis caused by all microorganisms led to septic shock, with the highest percentage seen in the *Pseudomonas* group (n = 330/94, 35.1% vs. n = 31,389/875,829, 3.5%, p < 0.00), followed by *S. aureus* (n = 339/1,004, 33.7% vs. n = 31,379/875,864, 3.5%, OR = 13.3, p = 0.00), and *E. coli* (2,149/6,439, 33.3 vs. 29,569/870,329, 3.3%, OR = 12.7, p = 0.00). Gastrointestinal bleeding was higher in patients with sepsis than those without sepsis, although the result was not significant (p > 0.05). The colon cancer patients who suffered from *S. aureus *sepsis were the highest who needed ventilators during hospitalization (34/1,004, 3.3% vs. 2,114/875,764, 0.2%, OR = 11, p = 0.00). The univariate and multivariate regression analyses for the complications are shown in Table 5.

**Table 5:** Complications prevalent in patients with and without sepsis categorized by organisms. AKI: acute kidney injury; GI: gastrointestinal

Organisms	With sepsis (N)	With sepsis (%)	Without colon cancer (N)	Without colon cancer (%)	Unadjusted odds ratio	P-value	Adjusted odds ratio	P-value
Methicillin-resistant *Staphylococcus aureus*								
AKI	1109	43	163,989	18	3.2	0.00	2.8	0.00
Peritonitis	39	1.5	9,354	1.0	1.4	0.3	1.3	0.4
Septic shock	624	24	31,094	3.5	8.6	0.00	7.6	0.00
GI bleed	184	7.1	56,549	6.4	1.1	0.5	1.0	0.7
Ventilator dependent	40	1.5	2,109	0.2	6.5	0.00	4	0.00
Staphylococcus aureus								
AKI	474	47	164,626	18.7	3.8	0.00	3.8	0.00
Peritonitis	20	2.0	9,374	1.0	1.8	0.3	1.8	0.3
Septic shock	339	33.7	31,379	3.5	13.7	0.00	13.3	0.00
GI bleed	75	7.4	56,659	6.4	1.1	0.5	1.0	0.7
Ventilator dependent	34	3.3	2,114	0.2	14	0.00	11	0.00
Escherichia coli								
AKI	3,109	48.2	161,989	18.7	4	0.00	4.1	0.00
Peritonitis	284	4.4	9,109	1.0	4.3	0.00	4.2	0.00
Septic shock	2,149	33.3	29,569	3.39	14	0.00	12.7	0.00
GI bleed	543	8.3	56,199	6.4	1.3	0.01	1.2	0.09
Ventilator dependent	79	1.2	2,069	0.2	5.2	0.00	4.4	0.00
Pseudomonas								
AKI	469	50	164,629	18.7	4.3	0.00	4.1	0.00
Peritonitis	25	2.6	9,369	1.0	2.5	0.04	2.3	0.05
Septic shock	330	35.1	31,389	3.5	14	0.00	12	0.00
GI bleed	60	6.3	56,674	6.4	0.9	0.9	0.8	0.6
Ventilator dependent	24	1.2	2,124	0.24	11	0.00	9.4	0.001
Enterococcus								
AKI	1,020	52	164,079	18	4.8	0.00	4.3	0.00
Peritonitis	90	4.6	9,304	1.0	4.5	0.00	4.2	0.00
Septic shock	544	28.1	31,174	3.5	10	0.00	8.9	0.00
GI bleed	140	7.2	56,595	6.4	1.1	0.5	1.0	0.9
Ventilator dependent	30	1.5	2,119	0.2	6.4	0.00	4.8	0.00
Other gram-negative organisms								
AKI	1,319	42	163,779	18.7	3.2	0.00	3.1	0.00
Peritonitis	65	2.1	9,329	1.0	1.9	0.03	1.8	0.06
Septic shock	1,010	33	30,709	3.5	13	0.00	12.2	0..00
GI bleed	180	5.9	56,554	6.4	0.8	0.5	0.8	0.6
Ventilator dependent	40	1.2	2,109	0.2	5.4	0.00	4.8	0.00

## Discussion

Our study highlights the prevalence of sepsis in patients admitted with colon cancer and suggests higher odds of mortality and disease-related complications associated with sepsis. It comprehensively analyzes baseline demographics, resource utilization, types of organisms involved, and outcomes in colon cancer patients who developed sepsis. In cancer patients, sepsis is one of the most common causes of admissions to intensive care units (ICUs) [12,13], and the leading cause of death in the United States [14]. The link between sepsis and colon cancer is complex and multifactorial.

The pathophysiology of sepsis in cancer patients is particularly complicated, as both conditions exhibit overlapping characteristics due to the ability of the host’s immune system to respond to an initial trigger effectively [9]. Immunosuppression increases the risk of severe infections, which are a common cause of mortality in this group [15]. Patients experience immunocompromised states due to various factors, including chemotherapy, radiotherapy, regular leukocyte activity disruptions, or corticosteroids.

Our analysis also highlights the organisms involved and their prevalence in cancer patients admitted with sepsis, the most common being *S. aureus* (n = 169/1,004, 17%), followed by *Pseudomonas* (n = 150/940, 16%), gram-negative organisms (n = 349/3,090, 11.2%), and *E. coli* (n = 214/1,935, 10%). Risk factors for *S. aureus* sepsis in colon cancer patients include healthcare-associated infections, immunosuppression, invasive devices such as central venous catheters, and repeated hospital admissions [16,17]. In one systematic review, 3% of the bloodstream infections were caused by MRSA [8]. In a systematic review and meta-analysis, Rojas et al. reported a 30-day mortality rate of 33.8% for community-onset bloodstream infections caused by *Pseudomonas aeruginosa* [18]. Ha et al. reported that cancer patients with bloodstream infections caused by extended-spectrum beta-lactamase (ESBL)-producing *E. coli* had a 30-day mortality rate of 22.1%, which was notably higher compared to non-ESBL *E. coli* infections (12.2%) [19]. Gram-negative organisms other than *E. coli* and *Pseudomonas* that can cause sepsis in colon cancer include *Klebsiella pneumonia*, *Enterobacter*, *Acinetobacter* species, and *Stenotrophomonas maltophilia* [20].

Our study showed a higher odds of mortality in a patient cohort who developed sepsis. A recent report revealed that in a cohort of over 1 million US patients hospitalized with sepsis, the mortality rate in the hospital was significantly higher among those with cancer-related sepsis compared to those with non-cancer-related sepsis (27.9% vs. 19.5%) [21]. Another study showed that the in-hospital mortality rate of cancer patients with sepsis ranges from 20% to 40% [22]. Breugom et al. showed that sepsis is linked to reduced overall survival in colon cancer patients, with a hazard ratio (HR) of 2.87 (95% confidence interval (CI) = 1.82-4.51) for one-year survival and an HR of 1.59 (95% CI = 1.25-2.04) for five-year survival [23].

Increased resource utilization, including increased length of hospital stay and total hospital charges, was also observed in our analysis. A study conducted among hospitalized cancer patients with severe sepsis showed findings similar to our research. Patients with severe sepsis and cancer had significantly longer hospital stays (7.1 days vs. 6.7 days, p < 0.0001) and higher costs ($27,400 vs. $8,700, p < 0.0001) compared to non-severe sepsis cancer hospitalizations [24].

Cancer patients with sepsis or septic shock require prompt treatment with a broad-spectrum antipseudomonal beta-lactam antibiotic, potentially combined with other agents effective against the suspected pathogens and appropriate for the injection site [25]. Before initiating empirical antibiotic therapy in cancer patients, several key factors should be considered, including previous infection with resistant organisms, other risk factors for antibiotic resistance, local epidemiology and resistance trends in the hospital, unit, and region, as well as patient-specific factors that may indicate a more complicated clinical course [9]. Due to increased antimicrobial resistance, antimicrobial stewardship is crucial for reducing overall antibiotic use and preventing the spread of resistance.

Strengths

Our study has several strengths. The NIS database captures a broad and diverse patient population. The study provides an in-depth look at baseline demographics, resource utilization, types of organisms involved, and patient outcomes in colon cancer patients admitted with sepsis. Using retrospective data from a national database offers insights into real-world clinical settings, capturing various hospital experiences across the United States.

Although recently, more studies are being done to understand and manage sepsis in patients with cancer, minimal data are available. To our knowledge, no study has explored the prevalence of sepsis and its impact on patients admitted with colon cancer. Although we highlight several vital associations, further prospective studies are required to better understand the pathophysiology of sepsis in cancer patients, particularly those with colon cancer.

Limitations

Our study has several limitations. We used the NIS database, which has limited information and can be affected by inaccuracies and missing data. Moreover, the study is retrospective and cannot establish a cause-effect relationship. The NIS is a sample of US hospitalizations and may only represent a part of the population, potentially limiting the generalizability of the findings. It also does not provide cancer-specific variables, such as cancer stage, treatment history, or progression, which could influence outcomes and sepsis severity.

## Conclusions

Our study highlights the prevalence of sepsis in colon cancer patients and its impact on outcomes. We observed that sepsis increases mortality, hospital stays, and healthcare costs. While the study provides valuable insights into the burden of sepsis in colon cancer, limitations such as the retrospective nature of the data and lack of cancer-specific variables warrant further investigation. Future prospective studies are needed to enhance understanding of the pathophysiology and management of sepsis in cancer patients, particularly in colon cancer.
